# Fisetin alleviates oxidative stress and promotes porcine early embryonic development via activation of the NRF2-ARE signalling pathway

**DOI:** 10.5713/ab.24.0691

**Published:** 2025-02-27

**Authors:** Hua-Kai Wei, Jia-Jia Qi, Yan-Qiu Wang, He-Xuan Qu, Chen-Xuan Yan, Tian-Tian Li, Yu Wang, Hao Sun, Bo-Xing Sun, Shuang Liang

**Affiliations:** 1 Department of Animals Sciences, College of Animal Sciences, Jilin University, Changchun, China

**Keywords:** Fisetin, NRF2-ARE, Oxidative Stress, Porcine Early Embryos

## Abstract

**Objective:**

We improved the developmental capacity of porcine early embryos via supplementation with fisetin during *in vitro* culture (IVC). In addition, we investigated the antioxidant mechanism of fisetin via activation of the NRF2-ARE signalling pathway in porcine early embryos.

**Methods:**

Fisetin (0, 1, 2.5 and 5 μM) was supplemented during IVC to observe its effects on the developmental ability of porcine parthenogenetic activation (PA), *in vitro* fertilization (IVF) and somatic cell nuclear transfer (SCNT) embryos. The effects of fisetin supplementation on the antioxidant capacity, mitochondrial function, cell proliferation and apoptosis levels of porcine PA embryos were detected via fluorescence staining, and the expression levels of genes related to apoptosis, pluripotency and the NRF2 pathway were also examined.

**Results:**

Compared with the control, 1 μM fisetin during IVC increased the developmental ability of porcine PA, IVF and SCNT embryos. Additionally, fisetin significantly decreased reactive oxygen species (ROS) and apoptosis levels; increased pluripotency during embryonic development, cell proliferation and glutathione levels; and improved mitochondrial function in PA embryos. Moreover, the levels of Kelch-like ECH-associated protein 1 (KEAP1) significantly decreased, and the levels of NFE2-like bZIP transcription factor 2 (NRF2) and its downstream antioxidant enzymes significantly increased after fisetin supplementation.

**Conclusion:**

Our data reveal that fisetin protects porcine early embryos from oxidative stress during IVC by activating the NRF2-ARE signalling pathway, thereby improving the success of *in vitro* embryo production.

## INTRODUCTION

High-quality embryos, a prerequisite for successful early embryonic development and subsequent postnatal development, constitute the material basis for the onset of life. However, in most instances, embryos obtained via *in vitro* culture (IVC) are usually of lower quality and quantity than those produced *in vivo* [1]. This phenomenon occurs due to a lack of physiological defence mechanisms, the presence of multiple potential sources of reactive oxygen species (ROS) or a lack of natural antioxidants to control basal ROS levels [2]. These differences may lead to a greater risk of oxidative stress *in vitro* than *in vivo*. Compared with other species, porcine embryos contain a large amount of energy-storing lipids and are more sensitive to the environment and susceptible to oxidative stress, thereby leading to a reduction in the quality of the embryos cultured *in vitro* [3]. Therefore, antioxidant supplementation may be beneficial for early embryonic development during IVC.

Numerous studies have demonstrated that flavonoids can maintain intracellular redox homeostasis not only by directly eliminating intracellular ROS but also by fortifying the cellular antioxidant defence system [4]. Fisetin is a flavonoid found in many vegetables, fruits and teas, especially in apples, strawberries, mangoes and onions [5]. It was previously shown to have various biological properties, such as antioxidant [6], anti-inflammatory [7], anticancer [8] and neuroprotective [9] properties. Fisetin is a well-studied antioxidant reported to protect against cell damage caused by oxidative stress by inhibiting ROS production [10,11], thereby maintaining the protective effect of the nonenzymatic defence system glutathione (GSH). Fisetin can increase the expression of antioxidant-related enzymes by activating the NFE2-like bZIP transcription factor 2 (NRF2)-mediated signalling pathway, thereby alleviating the oxidative stress caused by traumatic brain injury (TBI) [5,12].

NRF2 is a key transcription factor in cells that plays a central role in regulating the expression of many antioxidant genes [13–15]. Under normal physiological conditions, NRF2 binds to Kelch-like ECH-associated protein 1 (KEAP1), is ubiquitinated by the KEAP1-cul3 E3 ligase complex and is rapidly degraded by the 26S proteasome in the cytoplasm [16]. Thus, this rapid turnover keeps NRF2 at low levels and prevents NRF2 translocation to the nucleus [15]. Under oxidative stress conditions, critical modifications to the cysteine residues of KEAP1 cause a conformational change, leading to disruption of KEAP1-NRF2 binding and preventing the degradation of NRF2 [17]. At this point, NRF2 enters the nucleus and binds to antioxidant response elements (AREs) to initiate the transcription of downstream genes. This process is facilitated by cAMP-response element binding protein (CREB) and transcriptional activation [18]. Upon exposure to oxidative stressors, NRF2 activates the transcription of a series of antioxidant and phase II detoxifying enzymes, such as haem oxygenase 1 (HO-1), superoxide dismutase 1 (SOD1), catalase (CAT), glutathione peroxidase 4 (GPX4), glutamate-cysteine ligase catalytic subunit (GCLC), glutamate-cysteine ligase modifier subunit (GCLM), and NAD(P)H quinone dehydrogenase 1 (NQO1) [19–21], which quench ROS. To date, only a few studies have focused on the effects of fisetin on animal reproduction, particularly early embryonic development. Hence, we studied the NRF2-mediated antioxidant pathway to explore the effects of fisetin on porcine early embryonic development. The results of our study provide insights into the embryonic IVC system and a theoretical basis for increasing the quality of porcine early embryos.

Given the important role of antioxidants in the *in vitro* development of early embryos, we explored the effects of fisetin on the development of porcine embryos produced *in vitro* after parthenogenetic activation (PA), *in vitro* fertilization (IVF) and somatic cell nuclear transfer (SCNT). In addition, we evaluated the effects of fisetin on mitochondrial function, pluripotency, cell proliferation, apoptosis and the antioxidant capacity of porcine PA embryos and confirmed the antioxidant mechanism of fisetin in porcine PA embryos via the NRF2-ARE signalling pathway.

## MATERIALS AND METHODS

### Chemicals

All chemical reagents used in the study were purchased from Sigma-Aldrich (St. Louis, MO, USA) unless otherwise specified. Fisetin (purity: 98.39%; MedChemExpress, Shanghai, China) was dissolved in dimethyl sulfoxide (DMSO; MedChemExpress; Concentration of use<0.1%) and then diluted to the specific concentrations used in the experiment.

### Collection of porcine oocytes and *in vitro* maturation

Porcine ovaries were collected from slaughterhouses and delivered to the laboratory within 1–2 h in a normal saline solution of 0.9 wt.% NaCl at 37°C. The selected 3–8 mm follicles were aspirated with a 10 mL syringe to collect cumulus–oocyte complexes (COCs). After washing with preheated phosphate-buffered saline containing 0.1% polyvinyl alcohol (PBS-PVA) 3 times, COCs with homogeneous cytoplasm and at least three intact layers of surrounding cumulus cells were selected under a stereomicroscope (Stemi 305; ZEISS, Oberkochen, Germany). A total of 80–100 COCs were transferred to each well of a 4-well plate (JET BIOFIL, Guangzhou, China) containing 500 μL of medium. The components of the mature media used were tissue culture medium (TCM)-199 (TCM-199; Invitrogen, Carlsbad, CA, USA), epidermal growth factor (10 ng/mL), luteinizing hormone (10 IU/mL; NSHF, Ningbo, China), follicle-stimulating hormone (10 IU/mL; NSHF), sodium pyruvate (0.91 mM), follicular fluid (10%) and penicillin/streptomycin (1%). Finally, the cell medium was covered with 400 μL of mineral oil. The COCs were cultured in an incubator at 38.5°C with 5% CO_2_ and saturated humidity for 42 to 44 h.

After *in vitro* maturation, the COCs were digested with 0.1% hyaluronidase to remove the surrounding expanded cumulus crown cells. Oocytes with a uniform ooplasm and an excreted first polar body were subjected to subsequent experiments.

### Parthenogenetic activation, somatic cell nuclear transfer, *in vitro* fertilization and embryonic *in vitro* culture

PA, IVF and SCNT procedures were performed according to our previous study [22,23]. For PA, oocytes excreting polar bodies were activated twice at 120 V/mm direct-current pulses for 60 μs and then transferred to microdroplets of Cytochalasin B (HY-16928; MedChemExpress) to equilibrate for 3 to 5 h. For IVF, the use of fresh porcine semen and the density of spermatozoa were adjusted to 1×10^6^/mL. Oocytes excreting polar bodies and diluted spermatozoa were incubated together in an incubator for 6 h to fertilize the oocytes, after which spermatozoa attached to the surface of the oocytes were blown off by blowing in IVC solution. For SCNT, the nuclei of the oocytes were first removed, and then a single porcine fibroblast subjected to 48 h of starvation was inserted as a nuclear donor into the perivitelline space of the enucleated oocyte. The reconstructed oocytes were cycled 3 times for fusion under the parameters of 120 V/mm for 30 μs. They were placed in IVC solution and incubated in an incubator for 3 h to check and remove unfused embryos.

Following PA, SCNT and IVF, the embryos were washed in porcine zygote medium (PZM)-5. Approximately 50 embryos were then transferred to a 500 μL suspension containing bicarbonate-buffered PZM-5 and bovine serum albumin (4 mg/mL), which was covered with 400 μL of mineral oil. Fisetin was added to the IVC medium at final concentrations of 0, 1, 2.5 and 5 μM, and the cells were cultured continuously in a 4-well plate at 38.5°C, 5% CO_2_ and saturated humidity for 6 days without changing the medium during culture.

### Quantitative real-time polymerase chain reaction

Total RNA was extracted from porcine early embryos via the TRIzel (YY101; Epizyme, Shanghai, China) method. Next, MonScriptTM RTIII All-in-one Mix with dsDNase (MR05101M; Monad, Jiangsu, China) was used to reverse transcribe the total RNA into cDNA. Quantitative real-time polymerase chain reaction was performed using MonAmpTM ChemoHS qPCR Mix (MQ00401S; Monad) in a CFX Duet Real-Time PCR System (Bio-Rad, Hercules, CA, USA) according to the manufacturer’s instructions. The reaction conditions were 95°C for 10 min, 95°C for 10 s, 60°C for 10 s and 72°C for 30 s for a total of 40 cycles. Table 1 shows complete information on the qPCR primers used in this study, which were synthesized by Sangon Biotech, and *GAPDH* was used as a reference gene.

### EdU incorporation assay and Hoechst staining

Blastocyst proliferation was evaluated using a BeyoClick EdU-555 cell proliferation assay kit (C0075S; Beyotime, Shanghai, China). Briefly, porcine blastocysts that developed from PA embryos were incubated in the dark for 2 h at 10 μM EdU at 38.5°C in air with 5% CO_2_ and saturated humidity. After incubation, the samples were washed with PBS-PVA 3 times for 5 min each time and then fixed with 4% paraformaldehyde for 15 min. After fixation, the samples were washed with PBS-PVA 3 times and then permeated with immunostaining permeabilization buffer supplemented with saponin (P0095; Beyotime) for 10 min. Afterwards, the samples were fully washed with PBS-PVA, stained with azide 555 solution for 30 min, and washed with PBS-PVA 3 times. Afterwards, the blastocysts were placed on slides, 10 μg/mL Hoechst33342 was added, and the samples were incubated for 10 min in the dark. EdU-positive cells were observed under a fluorescence microscope and analysed via NIH ImageJ 1.8.0 software (National Institutes of Health, Bethesda, MD, USA).

### TUNEL assay

Blastocyst apoptosis was detected using a one-step TUNEL apoptosis assay kit (MA0223; MeilunBio, Dalian, China). Briefly, porcine blastocysts were first fixed in 4% paraformaldehyde for 1 h and then washed 3 times with PBS-PVA. The samples were then permeated in immunostaining permeabilization buffer with saponin (P0095; Beyotime) for 10 min, washed with PBS-PVA 3 times and incubated at 37°C with TUNEL assay solution for 1 h. Then, the incubated blastocysts were washed 3 times with PBS-PVA and evenly placed on slides, and an appropriate amount of 10 μg/mL Hoechst33342 was added to the slides and incubated for 10 min in the dark. The total number of apoptotic nuclei in the porcine blastocysts was analysed via ImageJ software, and apoptosis was evaluated according to the percentage of apoptotic nuclei in the blastocysts.

### Immunofluorescence

Porcine blastocysts that developed to day 6 were fixed with 4% paraformaldehyde for 30 min at room temperature and then washed 3 times with PBS-PVA for 5 min/wash, followed by 3 washes with PBS-PVA for each subsequent operation. Permeabilization was performed via incubation with Enhanced Immunostaining Permeabilization Buffer (P0097; Beyotime) for 30 min at room temperature. The incubator was closed for 1 h using QuickBlock Blocking Buffer for Immunol Staining (P0260; Beyotime) for 1 h in an incubator. NRF2 (1:300, 16396-1-AP; Proteintech, Wuhan, China) and KEAP1 (1:300, 60027-1-Ig; Proteintech) were both mixed, and small drops were made into which the embryos were transferred and incubated overnight at 4°C; Cy3-conjugated Goat Anti-Rabbit IgG (H+L) (1:100, SA00009-2; Proteintech) and Fluorescein-conjugated Goat Anti-Mouse IgG (H+L) (1:100, SA00003-1; Proteintech) were mixed, and the embryos were transferred to the samples, which were incubated at room temperature for 1 h. Finally, the incubated blastocysts were evenly placed on slides, and an appropriate amount of 10 μg/mL Hoechst33342 was added to the slides and incubated for 10 min in the dark. The signal intensities were observed under a confocal microscope (Zeiss), and the fluorescence intensities were quantified via ImageJ software.

### Western blotting

Embryos that developed to day 6 (n = 80/per replicate) were collected for WB analysis. The prepared embryo samples were treated with a lysis buffer composed of ddH_2_O, 0.5 mM Tris-HCl, 50% glycerol, 10% sodium dodecyl sulfate, bromophenol blue and β-mercaptoethanol. The protein was extracted at 95°C for 10 min. After protein denaturation, Omni-Easy One-step Colour polyacrylamide gel electrophoresis Gel (PG212 or PG213; Epizyme) with an appropriate molecular weight was selected to separate the protein samples in electrophoretic buffer, and then the proteins were transferred to a polyvinylidene fluoride membrane (PVDF; IPVH000101, Immobilon; Merck, Darmstadt, Germany). The PVDF membrane was blocked with protein-free rapid blocking buffer (PS180P; Epizyme) at room temperature for 30 min and washed with TBST 3 times for 10 min each, followed by 3 washes for 10 min with TBST for each subsequent operation. After incubation with the primary antibody at 4°C overnight. The membrane was subsequently incubated with the secondary antibody for 1 h at room temperature. After sufficient Omni-ECL Femto Light Chemiluminescence reagent (SQ201; Epizyme) was added to the PVDF membrane, images of the protein bands were captured with a Tanon5200 imaging system (Tanon, Shanghai, China). The immunoblots were analysed with ImageJ software. Please refer to Table 2 for complete information on the antibodies used in this study.

### Reactive oxygen species and glutathione staining

To determine the intracellular ROS and GSH levels, embryos that developed to the 4 cell stage and blastocyst stage were separately treated with a ROS assay kit (S0033S; Beyotime) and CellTracker fluorescent probes (C12881; Thermo Fisher Scientific, Waltham, MA, USA). The embryos were incubated in PBS-PVA containing 10 μM 2′,7′-DCFH or 10 μM 4-CMF2HC for 15 or 30 min, respectively. After the incubated embryos were washed three times in PBS-PVA, the fluorescence signals of ROS and GSH were captured in jpg format via a digital camera connected to a fluorescence microscope, and the fluorescence intensity was analysed via ImageJ software.

### Mitochondrial membrane potential assay

The mitochondrial membrane potential (MMP) was determined in 4 cell stage and blastocyst stage PA embryos by MitoTracker Red CMXRos staining. Following the manufacturer’s guidelines, MitoTracker Red CMXRos dye (C1035; Beyotime) was used to dilute the solution to 200 nM, and the embryos were incubated at 37°C for 30 min. After 3 washes with PBS-PVA, red fluorescence TIFF-formatted images were observed and captured via fluorescence microscopy, and the fluorescence intensity was analysed via ImageJ software.

### Adenosine triphosphate level measurements

The adenosine triphosphate (ATP) levels of porcine 4 cell stage and blastocyst-stage PA embryos were determined via an enhanced ATP assay kit (S0027; Beyotime). According to the instructions, 80 to 120 porcine embryos were collected, 200 μL of ATP lysis buffer was added to fully lyse the cells, the cells were centrifuged at 12,000×g for 5 min at 4°C, and the supernatant was collected for subsequent analysis. ATP test diluent was added to the ATP test reagent at a ratio of 4:1 to prepare the ATP test working liquid. Then, 100 μL of ATP detection reagent was added to a 96-well plate and incubated for 3 to 5 min to consume the ATP substrate, after which 20 μL of the standard solution was added. The mixture was thoroughly mixed and immediately tested. The emitted light signal was measured with a microplate reader (Tecan, Shanghai, China).

### Molecular docking

The binding of fisetin to the KEAP1 protein was evaluated using molecular docking analysis. The molecular structure of fisetin (PubChem CID: 5281614) was retrieved from the PubChem database (https://pubchem.ncbi.nlm.nih.gov/), and the 3D molecular structure of the KEAP1 (PDB ID: 7K2A) protein was retrieved from the PDB database (https://www.rcsb.org/). According to a previous standard protocol [24], several essential steps, such as molecular force field optimization, the addition of hydrogens, the deletion of water molecules, and the elimination of unrelated protein chains and proto-ligands from protein structures, are needed. After the ligand was optimized, semiflexible docking was used for docking, and images were prepared via PyMOL version 2.3.0. AutoDock Vina 1.2.0 was used for molecular docking to obtain the docking binding free energy as well as the docking result file.

### Statistical analysis

At least three independent biological replicates were used for each experiment. The results were first normalized for fluorescent staining, mRNA, protein and ATP. Then, GraphPad 9.5.0 software (GraphPad, San Diego, CA, USA) was used to evaluate the normal distribution of the data, and then the ANOVA and an independent sample t test were used for the statistical analysis of the data that passed the Shapiro–Wilk test. The Mann‒Whitney test was used for the statistical analysis of data that did not pass the Shapiro‒Wilk test. The data are expressed as the mean±standard error, and p<0.05 was considered to indicate statistical significance.

## RESULTS

### Fisetin promoted porcine parthenogenetic activation embryo development and increased blastocyst quality

**Figure 1:**
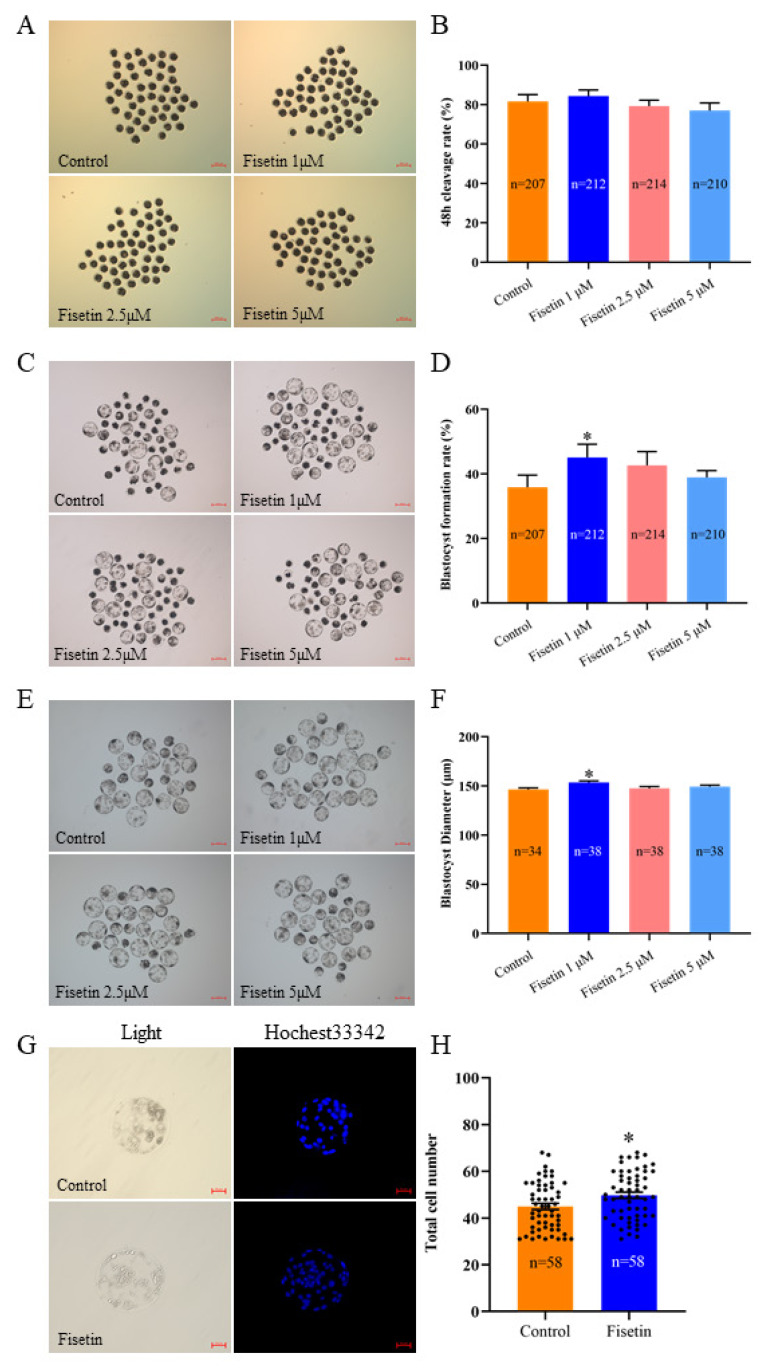
Fisetin promoted the development of porcine parthenogenetic activation (PA) embryos. (A, C) Representative images of porcine PA embryonic development at 48 h and on day 6 (6 d) in the control and different concentrations of fisetin groups; scale bar = 200 μm. (B, D) 48 h cleavage rates and 6 d blastocyst formation rates of porcine PA embryos. (E) 6 d blastocyst images obtained after supplementation with different concentrations of fisetin; scale bar = 200 μm. (F, H) Average diameter and total cell number in blastocysts. (G) Representative fluorescence images of Hoechst33342-stained blastocysts; scale bar = 50 μm. The data are expressed as the mean±SEM of at least three independent experiments. Asterisks above the bars indicate significant differences from the control group (* p<0.05). SEM, standard error of the mean.

To determine the optimal concentration of fisetin, the IVC medium was supplemented with 0, 1, 2.5, or 5 μM fisetin. We found that the 48 h cleavage rate of embryos treated with 1 μM fisetin increased from 81.59% to 84.36% (Figures 1A, 1B; 84.36±6.06% vs. 81.59±3.47%; p>0.05), and the blastocyst formation rate increased from 35.85% to 45.06% (Figures 1C, 1D; 45.06±8.23% vs. 35.85±7.50%; p<0.05) compared with the control group. Additionally, the blastocyst diameter in the 1 μM fisetin group was significantly greater than that in the control group (Figures 1E, 1F; 154.06±1.57 vs. 146.46±1.53; p<0.05). On the basis of the above results, 1 μM was selected as the optimum concentration of fisetin, and subsequent experiments were conducted with the control and 1 μM fisetin groups. The number of blastocysts increased significantly after fisetin treatment (Figures 1G, 1H; 49.72±1.35 vs. 44.90±1.35; p<0.05). The results showed that supplementation with fisetin during IVC promoted the development of porcine PA embryos.

### Fisetin improved the developmental potential of porcine *in vitro* fertilization and SCNT embryos

**Figure 2:**
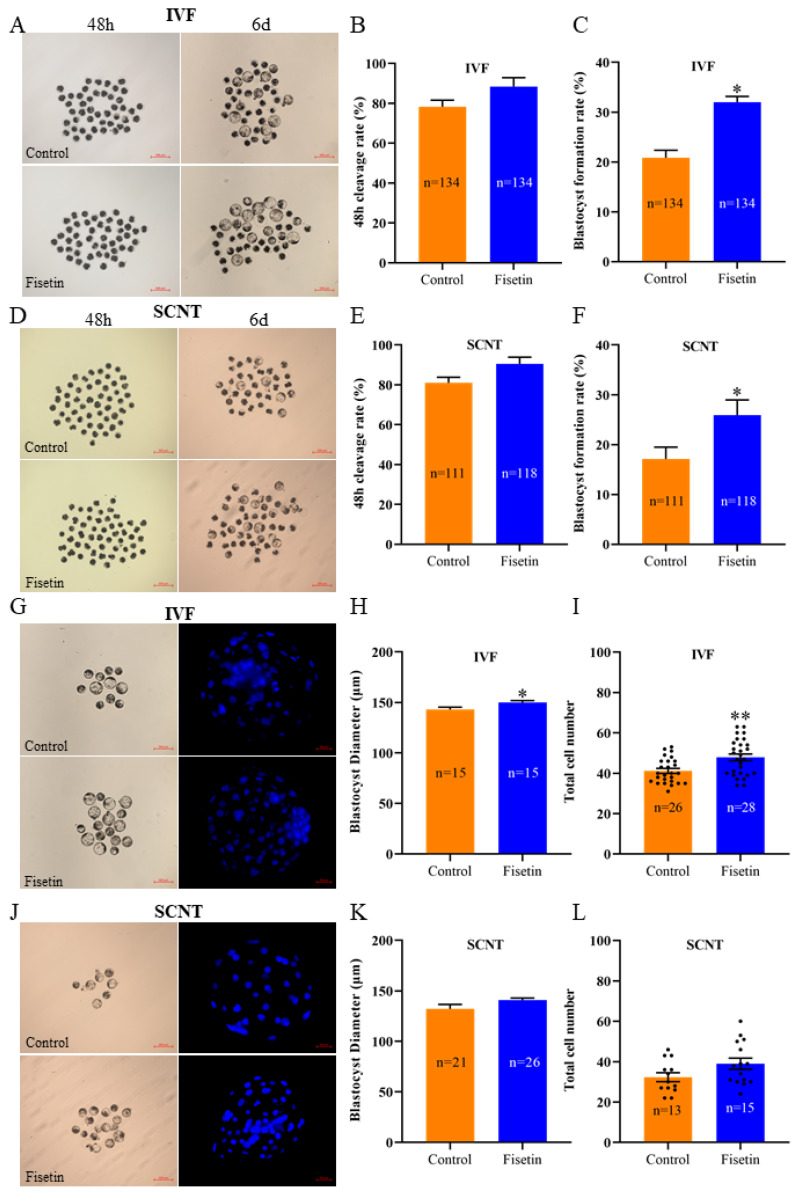
Fisetin promoted the development of porcine *in vitro* fertilization (IVF) and somatic cell nuclear transfer (SCNT) embryos. (A, D) Representative images of porcine IVF and SCNT embryonic development at 48 h and day 6 (6 d) in the control and fisetin groups; scale bar = 500 μm. (B, C, E, F) 48 h cleavage rates and 6 d blastocyst formation rates of porcine IVF and SCNT embryos. (G, J) Representative blastocyst and fluorescence images of Hoechst33342-stained porcine IVF and SCNT blastocysts; scale bar = 50/500 μm. (H, I, K, L) Average diameter and total cell number in porcine IVF and SCNT blastocysts. The data are expressed as the mean±SEM of at least three independent experiments. Asterisks above the bars indicate significant differences from the control group (* p<0.05, ** p<0.01). SEM, standard error of the mean.

Although PA embryos are commonly used as models for *in vitro* embryo development studies, it has different characteristics than IVF and SCNT embryos do. Therefore, we evaluated the effects of fisetin treatment on the formation and quality of blastocysts in porcine IVF and SCNT embryos. Compared with that of the control group, the blastocyst formation rate of fisein treated IVF embryos increased from 20.84% to 31.98% (Figures 2A, 2C; 31.98±1.17% vs. 20.84±1.54%; p<0.05), and SCNT embryos increased from 17.12% to 25.92% (Figures 2D, 2F; 25.92±3.05% vs. 17.12±2.38%; p<0.05), both of which significantly increased, whereas the 48 h cleavage rate increased but not significantly (Figures 2A, 2B, 2D, 2E; 88.37± 4.57% vs. 78.30±3.31%; 90.52±3.39% vs. 81.08±2.70%; p>0.05). In addition, after the embryos were treated with fisetin, the blastocyst diameter and the number of blastocyst cells of the IVF embryos significantly increased (Figures 2G–2I; 150.02± 1.71 vs. 143.03±2.33; 47.86±1.68 vs. 42.82±1.62; p<0.05, p<0.01). The differences in blastocyst diameter and the number of blastocyst cells were not significant among the SCNT embryos (Figures 2J–2L; 140.90±1.98 vs. 132.12±4.38, 41.00±3.01 vs. 33.82±2.35; p>0.05). The results indicated that fisetin supplementation during IVC could also promote the development of porcine IVF and SCNT embryos, which is consistent with the results obtained for PA embryos.

### Fisetin enhanced the pluripotency of porcine parthenogenetic activation blastocysts

**Figure 3:**
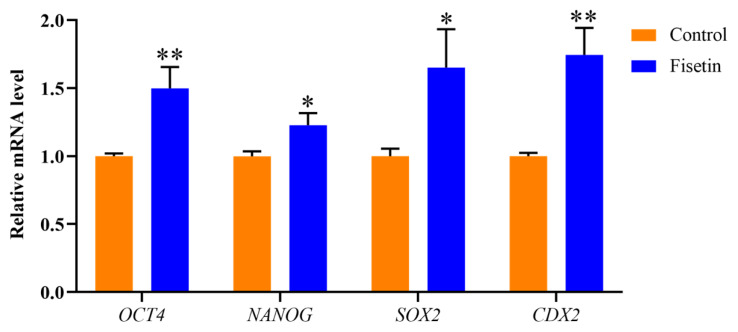
Fisetin increased the pluripotency of porcine parthenogenetic activation (PA) blastocysts. Relative expression of the pluripotency genes *OCT4*, *NANOG*, *SOX2* and *CDX2* in porcine PA blastocysts. The data are expressed as the mean±SEM of at least three independent experiments. Asterisks above the bars indicate significant differences from the control group (* p<0.05, ** p<0.01). SEM, standard error of the mean.

The normal expression of pluripotent genes can ensure the normal development and differentiation of embryos and is also an important index for measuring the quality of blastocysts. Compared with those in the control group, the mRNA levels of the *Octamer-binding transcription factor (OCT4*; p<0.01), *Nanog homeobox (NANOG*; p<0.05), *SRY-box transcription factor 2 (SOX2*; p<0.05) and *Caudal type homeobox 2 (CDX2*; p<0.01) genes related to pluripotency during embryonic development were upregulated to varying degrees (Figure 3). The results showed that fisetin enhanced the pluripotency of porcine PA embryos.

### Fisetin promoted cell proliferation and inhibited apoptosis in porcine parthenogenetic activation blastocysts

**Figure 4:**
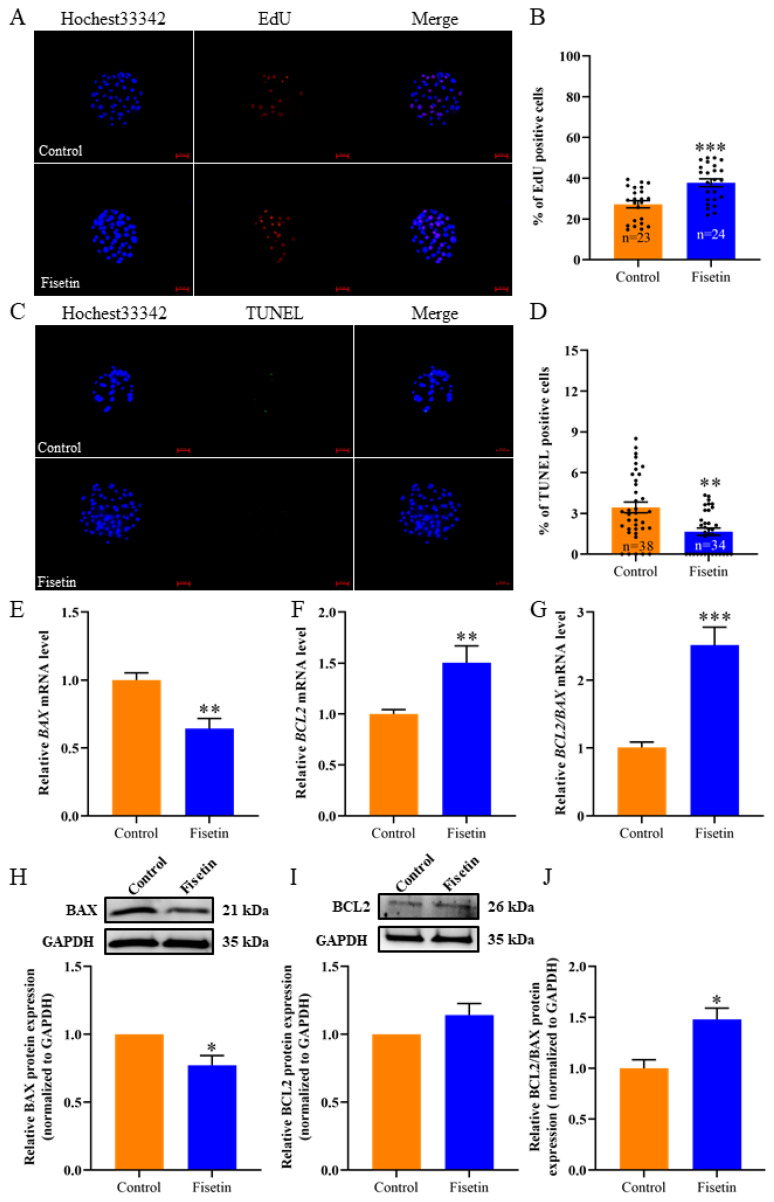
Fisetin increased cell proliferation and decreased apoptosis in porcine parthenogenetic activation (PA) blastocysts. (A, C) Representative images of EdU-positive cells and TUNEL-positive cells detected in porcine PA blastocysts; scale bar = 50 μm. (B, D) Percentage of EdU-positive cells and the apoptosis rate in porcine PA blastocysts. (E–G) Relative expression of *BAX*, *BCL2* and *BCL2*/*BAX* mRNA. (H–J) Relative protein expression of BAX, BCL2 and BCL2/BAX. The data are expressed as the mean±SEM of at least three independent experiments. Asterisks above the bars indicate significant differences from the control group (* p<0.05, ** p<0.01, *** p<0.001). SEM, standard error of the mean.

EdU staining revealed that fisetin strongly increased cell proliferation in porcine PA embryos (Figures 4A, 4B; 37.76± 1.91% vs. 27.18±1.75%; p<0.001), and TUNEL staining revealed that fisetin significantly decreased apoptosis in porcine PA embryos (Figures 4C, 4D; 1.66±0.26% vs. 3.67±0.45%; p<0.01). Moreover, fisetin significantly reduced the mRNA and protein levels of BAX (Figures 4E, 4H; p<0.01, p<0.05), and the mRNA levels of *BCL2* and *BCL2/BAX* were increased (Figures 4F, 4G; p<0.01, p<0.001); The protein levels of BCL2/BAX were increased (Figure 4J; p<0.05), but the protein levels of BCL2 didn’t significantly change (Figure 4I; p>0.05). The results showed that fisetin can increase the proliferation and inhibit the apoptosis of cells in porcine PA embryos.

### Fisetin increased antioxidant capacity and improved mitochondrial function in porcine parthenogenetic activation embryos

**Figure 5:**
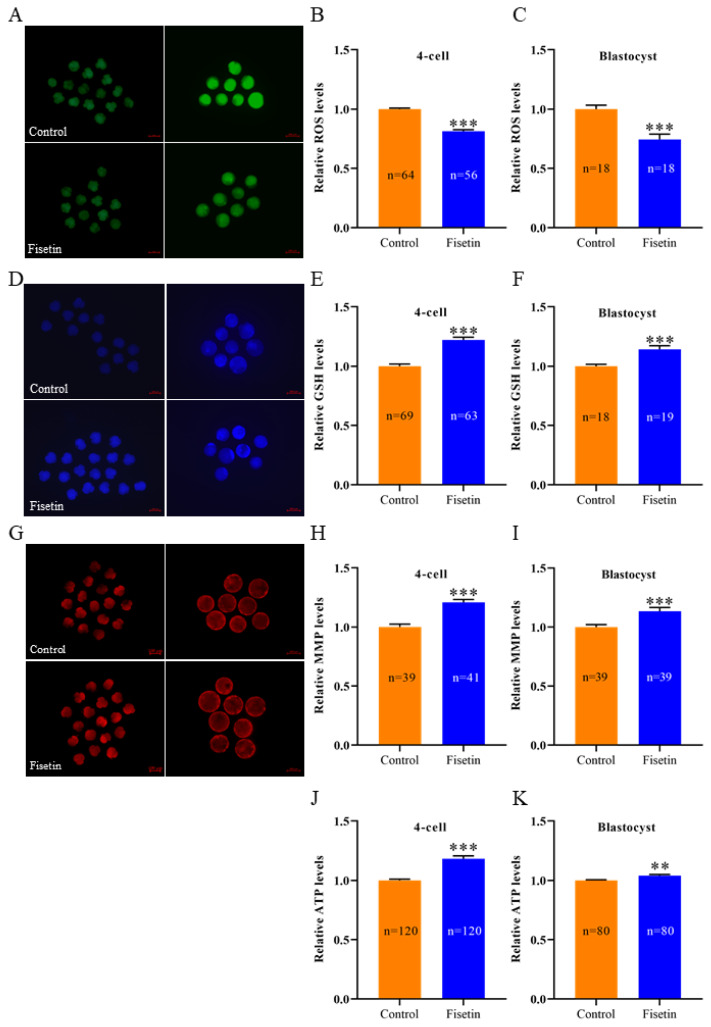
Fisetin increased the antioxidant capacity and mitochondrial function in porcine parthenogenetic activation (PA) embryos. (A, D, G) Representative fluorescence images of 4 cell and blastocyst 2′,7′-DCFH, 4-CMF2HC and MitoTracker Red CMXRos staining of porcine PA embryos; scale bar = 100/200 μm. (B, C, E, F, H–K) Intracellular relative reactive oxygen species (ROS), glutathione (GSH), Mitochondrial membrane potential (MMP) and Adenosine triphosphate (ATP) levels during the 4 cell and blastocyst stages in porcine PA embryos. The data are expressed as the mean±SEM of at least three independent experiments. Asterisks above the bars indicate significant differences from the control group (** p<0.01, *** p<0.001). SEM, standard error of the mean.

Excessive ROS accumulation can cause oxidative stress in porcine early embryos and cause mitochondrial dysfunction. Therefore, we examined the ROS, GSH, MMP and ATP levels in the 4 cell stage and blastocyst stage of porcine PA embryos. The results revealed that the ROS levels were significantly lower (Figures 5A–5C; p<0.001), and the GSH levels were significantly greater (Figures 5D–5F; p<0.001) in the fisetin group compared with the control group. In addition, the MMP was significantly elevated (Figures 5G–5I; p<0.001), and the mitochondrial ATP levels were significantly increased (Figures 5J, 5K; p<0.001, p<0.01) in the fisetin group. The results indicated that fisetin increased the antioxidant capacity and improved mitochondrial function in porcine PA embryos. Fisetin is a natural compound with excellent antioxidant effects, and our results demonstrated that fisetin increased the antioxidant capacity and improved the mitochondrial function of porcine PA embryos, which may be the main role of fisetin in the IVC of early porcine embryos.

### Fisetin reduces the risk of oxidative stress in porcine parthenogenetic activation embryos via the NRF2-ARE signalling pathway

**Figure 6:**
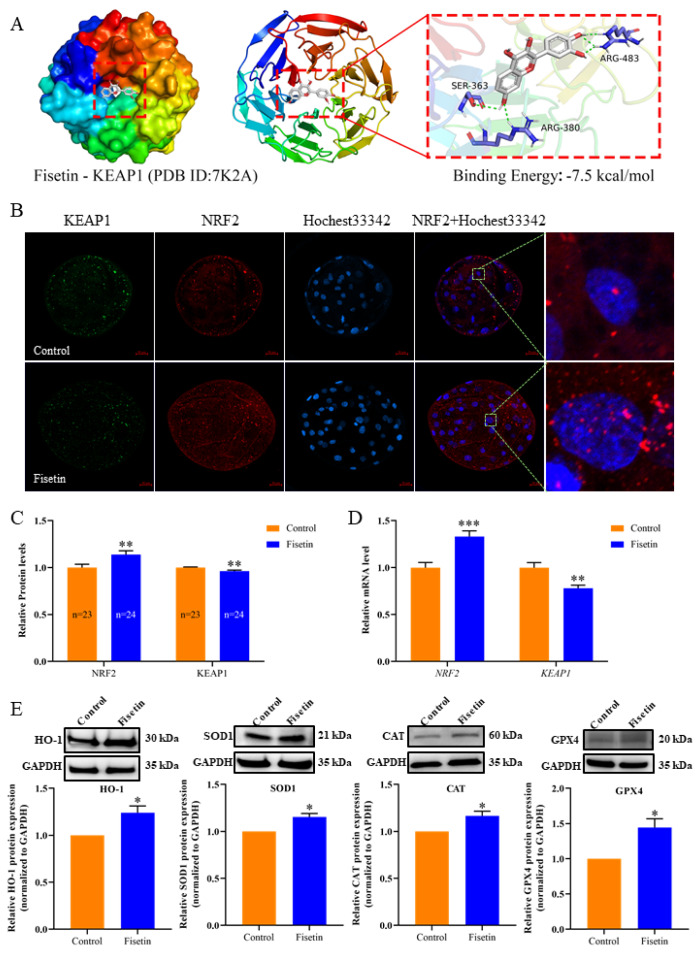
Fisetin activates the NRF2-ARE signalling pathway. (A) The molecular docking of fisetin with the KEAP1 protein can be viewed as follows: the colourful protein is KEAP1, the small grey molecule is fisetin, and the small blue molecule is an amino acid residue in the protein that forms an interaction with fisetin. The force binding mode represented by the green dashed line is conventional hydrogen bonding. (B) Representative immunofluorescence images of KEAP1, NRF2, Hoechst33342 and Merge of NRF2+Hoechst33342 of porcine PA blastocysts; scale bar =50 μm. (C) Quantification of the fluorescence intensity of the NRF2 and KEAP1 proteins in porcine PA blastocysts. (D) Relative mRNA expression of *NRF2* and *KEAP1* in porcine PA blastocysts. (E) Relative protein expression of the antioxidant-related enzymes HO-1, SOD1, CAT and GPX4 in porcine PA blastocysts. The data are expressed as the mean±SEM of at least three independent experiments. Asterisks above the bars indicate significant differences from the control group (* p<0.05, ** p<0.01, *** p<0.001). HO-1, haem oxygenase 1; SOD1, superoxide dismutase 1; CAT, catalase; GPX4, glutathione peroxidase 4; SEM, standard error of the mean.

Studies have shown that the NRF2-ARE signalling pathway is the main pathway by which fisetin exerts its antioxidant effects [12]. We first performed molecular docking prediction of fisetin and the KEAP1 protein. The results revealed that fisetin has multiple binding sites for KEAP1 and a binding energy of -7.5 kcal/mol (Figure 6A), indicating that fisetin likely affects the structure and bioactivity of the KEAP1 protein. To further determine the relationship between fisetin and the NRF2-ARE signalling pathway, we detected NRF2 and KEAP1 via IF and found that the level of NRF2 was significantly elevated and that of KEAP1 was significantly decreased in the fisetin group (Figures 6B, 6C; p<0.01), and also the mRNA of *NRF2* was significantly increased and *KEAP1* was significantly decreased (Figure 6D; p<0.001, p<0.01). The combination of qPCR and WB revealed that the levels of the NRF2 downstream related antioxidant enzymes HO-1, SOD1, CAT and GPX4 were significantly elevated (Figure 6E; p<0.05), as did the mRNA levels of *GCLC* (p<0.01), *GCLM* (p<0.05) and *NQO1* (p<0.001) (Supplement 1). These results indicate that fisetin improves the antioxidant capacity of porcine early embryos via activation of the NRF2-ARE signalling pathway.

## DISCUSSION

**Figure 7:**
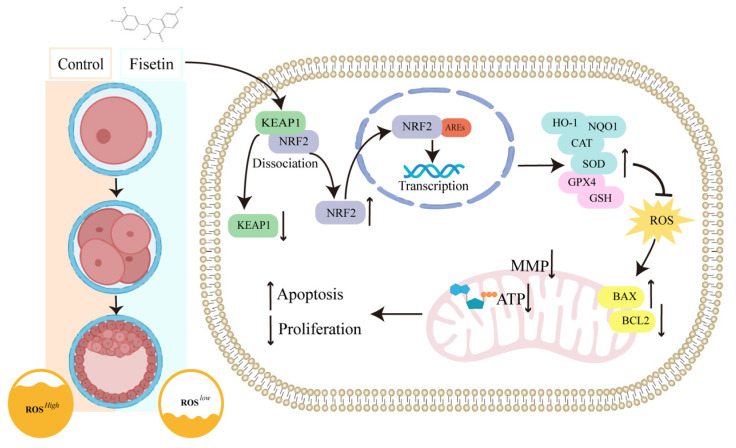
This schematic shows that fisetin promotes the expression of phase II antioxidant enzymes by interfering with the protein of KEAP1, causing the NRF2 protein to dissociate from it, increasing NRF2 levels and binding to AREs in the nucleus. Subsequently, mitochondrial function is improved, and cell proliferation capacity and pluripotency are enhanced, along with reduced apoptosis, thereby promoting porcine early embryonic development. AREs, antioxidant response elements; HO-1, haem oxygenase 1; NQO1, NAD(P)H quinone dehydrogenase 1; CAT, catalase; SOD, superoxide dismutase; GPX4, glutathione peroxidase 4; GSH, glutathione; ROS, reactive oxygen species; MMP, mitochondrial membrane potential; ATP, adenosine.

Early embryonic development, which requires a large amount of energy, is a critical period for new life. Mitochondrial oxidative phosphorylation generates more than 90% of all ATP. [25,26]. Moreover, mitochondrial oxidative phosphorylation of ATP is produced along with the generation of ROS [27]. *In vivo*, antioxidants in follicular and oviductal fluids protect embryos from oxidative stress, whereas *in vitro*, embryos rely on their own antioxidant defence mechanisms, including antioxidant enzymes and the nonenzymatic defence system GSH, to prevent oxidative stress [28]. Because oxidative stress plays a critical role in early embryonic development, we explored whether fisetin protects early embryonic development by inhibiting oxidative stress. However, the quality of porcine *in vitro*-produced embryos still falls short in comparison with that of in *vivo*-derived embryos [29], so it is crucial to continue to search for effective antioxidants, improve the IVC system, and explore its use for *in vitro* production of early porcine embryos. In this study, we investigated the effects of fisetin on porcine early embryo development and the potential underlying mechanisms involved. Our results revealed that fisetin inhibits oxidative stress via activation of the NRF2-ARE signalling pathway, which promotes the development of porcine early embryos (Figure 7).

Previous studies have proposed that fisetin, a known antioxidant, abrogates oxidative stress and augments the endogenous antioxidant defence system. On the one hand, fisetin acts as an ROS scavenger, reducing ROS production [30]; on the other hand, fisetin can affect the activity of oxidant enzymes such as xanthine oxidase and nicotinamide adenine dinucleotide phosphate (NADPH) oxidase, preventing excessive ROS production [31]. In this study, fisetin reduced intracellular ROS levels and increased GSH levels after IVC. Numerous studies using zebrafish [32], H9c2 cells [33] and bovine endometrial epithelial cell lines [34] have shown that fisetin can increase the cellular antioxidant capacity during *in vitro* induction culture. A reduced MMP and compromised mitochondrial function are indicators of ROS accumulation and apoptosis in early embryos [35], which account for their developmental retardation. Additionally, reports indicate that the NRF2/ARE transcriptional pathway is the master regulator of the response to mitochondrial dysfunction. NRF2 has beneficial effects on mitochondrial biogenesis and function [36]. Our data showed that fisetin supplementation effectively alleviated mitochondrial dysfunction by eliminating excess ROS, thereby inhibiting apoptosis.

Excessive ROS accumulation can induce cellular damage and apoptosis, which may lead to embryonic development arrest [28]. In the present study, we found that fisetin significantly increased the expression of genes related to pluripotency, the diameter of blastocysts and the number of blastocysts in porcine early embryos. We then wanted to determine whether the increase in cell proliferation and decrease in apoptosis induced by fisetin supplementation would increase the quality of porcine early embryos. Fisetin effectively reduces the corticosterone-mediated generation of ROS and inhibits corticosterone-induced cell death in PC12 cells [37]. Furthermore, other empirical studies have shown that fisetin suppresses neuronal cell death and apoptosis, increases the expression of BCL2, and decreases the expression of BAX and caspase-3 after TBI [12]. Similarly, our findings revealed that fisetin treatment increased cell proliferation and decreased apoptosis in porcine early embryos.

Previous research has reported a protective role of NRF2 in porcine parthenote embryos, particularly in embryos cultured under metabolically stressful conditions [38]. Many subsequent studies have demonstrated that NRF2 is activated and plays a protective role in mammalian embryonic development. For example, luteolin-mediated activation of the NRF2/KEAP1 signalling pathway contributes to the increased production of porcine embryos with high developmental competence [39]. Moreover, maternal exposure to hyperbaric oxygen at the early stage upregulated NRF2-NOTCH1-CDX2 expression, thereby inducing apoptosis and impairing inner cell mass specification [40]. We speculate that fisetin mitigates oxidative stress in porcine early embryos via activating the NRF2-ARE signalling pathway.

To verify our hypothesis, we first predicted the binding of fisetin to KEAP1 using molecular docking and then examined the associated proteins and mRNAs. Our results are consistent with the speculation that the mRNA and protein expression levels of KEAP1 were significantly downregulated, combined with the results of molecular docking of fisetin with KEAP1, indicating that fisetin may prevent proteasomal degradation of the NRF2 protein by interfering with the KEAP1-dependent E3 ubiquitin activity, allowing NRF2 to translocate to the nucleus and resulting in increased accumulation of NRF2 in the nucleus. This finding is consistent with previous reports that fisetin promotes the translocation of NRF2 from the cytoplasm to the nucleus, thereby enhancing its ability to bind to AREs [12]. Moreover, the expression of the antioxidant enzymes HO-1, GPX4, SOD1 and CAT downstream of the NRF2-ARE pathway was significantly elevated, and the mRNAs of *GCLC*, *GCLM* and *NQO1* were significantly upregulated. However, the detailed mechanisms by which fisetin activates the NRF2 pathway have not been fully elucidated and need to be investigated in future studies.

## CONCLUSION

In this study, we demonstrated for the first time that fisetin was able to protect early porcine embryos by activating the NRF2-ARE signalling pathway against oxidative stress. These results may provide a promising strategy for optimizing the porcine IVC system and provide insights into the role of the NRF2-ARE signalling pathway in porcine early embryogenesis. Our findings can provide a theoretical basis for improving the yield of porcine embryos produced *in vitro* and for preserving endangered porcine breeds.

## Figures and Tables

**Table 1:** Primer sequences for qPCR

Gene	Forwards (5′–3′)	Reverse (5′–3′)	Gene Bank
*BAX*	GCTTCAGGGTTTCATCCA	TGTCCAGTTCATCTCCAAT	XM_003127290.5
*BCL2*	GAACTGGGGGAGGATTGTGG	CATCCCAGCCTCCGTTATC	XM_021099593.1
*CDX2*	AGAACCGCAGAGCGAAGG	AGAACCCCAGGGACAGAG	NM_001278769.1
*GAPDH*	TCGGAGTGAACGGATTTGGC	TGACAAGCTTCCCGTTCTCC	NM_001206359.1
*GCLC*	GCCCAACCCCGTGGAAGA	ACGCCCCAGCGACAATCA	XM_021098556.1
*GCLM*	GCACAGGTAAAACCAAACA	GCTCTTAACAATGACCGAGT	XM_001926378.4
*NANOG*	AGCGAATCTTCACCAATG	GCTTGTGGAAGAATCAGG	NM_001129971.2
*NQO1*	CCAGCAGCCCGGCCAATCTG	AGGTCCGACACGGCGACCTC	NM_001159613.1
*OCT4*	GTGAGAGGCAACCTGGAGAG	TCGTTGCGAATAGTCACTGC	NM_001113060.1
*SOX2*	CGGCAACCAGAAGAACAG	CTCCGTCTCCGACAAAAG	NM_001123197.1

qPCR, quantitative real-time polymerase chain reaction.

**Table 2:** Antibodies for Western blot analysis

Antibody name	Working concentration	Catalog number	Supplier
BAX	1: 1000	50599-2-Ig	Proteintech
BCL2	1: 1000	68103-1-Ig	Proteintech
CAT	1: 1000	66765-1-Ig	Proteintech
GAPDH	1: 10000	60004-1-Ig	Proteintech
GPX4	1: 1000	DF6701	Affinity
HO-1	1: 1000	10701-1-AP	Proteintech
SOD1	1: 1000	67480-1-Ig	Proteintech
Anti-mouse secondary antibody	1: 8000	SA00001-1	Proteintech
Anti-rabbit secondary antibody	1: 10000	SA00001-2	Proteintech

## SUPPLEMENTARY MATERIAL

**Supplement 1**. Relative expression of *GCLC*, *GCLM* and *NQO1* mRNAs in porcine PA blastocysts
