# Supplementary material for: Fisetin alleviates oxidative stress and promotes porcine early embryonic development via activation of the NRF2-ARE signalling pathway
Source: Anim Biosci. 2025 Feb 27;38(6):1160–74. doi: 10.5713/ab.24.0691 (PMC12061573; doi:10.5713/ab.24.0691)
Supplement: Supplementary file 1 [file ab-24-0691-Supplementary-1.pdf]

Figure legend

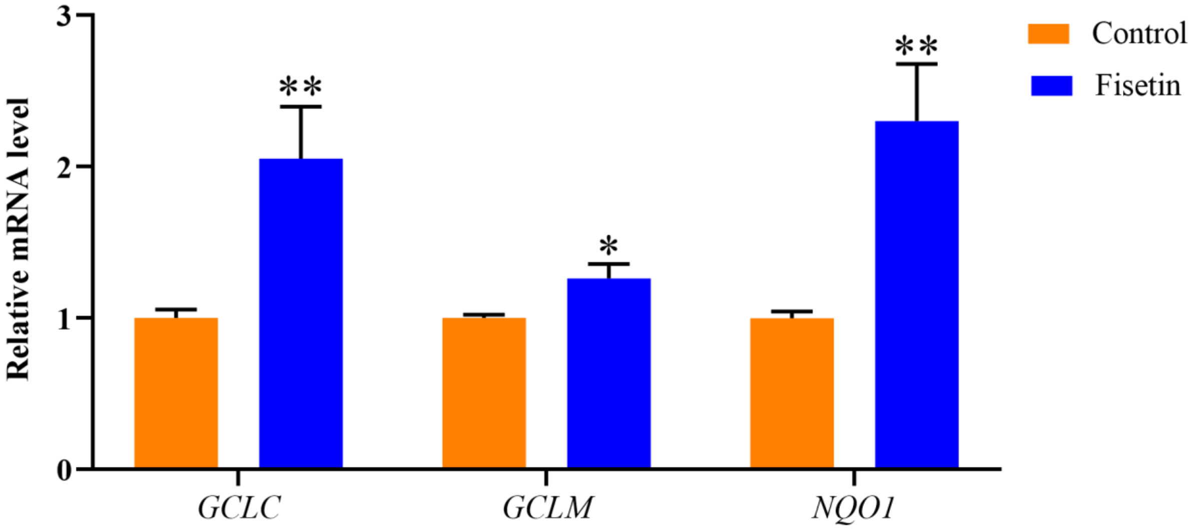

Supplement 1. Relative expression of *GCLC*, *GCLM* and *NQO1* mRNAs in porcine PA blastocysts.
